# Supplementary material for: Causal inferences and real-world evidence: A comparative effectiveness evaluation of abiraterone acetate against enzalutamide
Source: PLoS One. 2023 Oct 26;18(10):e0293000. doi: 10.1371/journal.pone.0293000 (PMC10602359; doi:10.1371/journal.pone.0293000)
Supplement: S1 Text — (DOCX) [file pone.0293000.s001.docx]

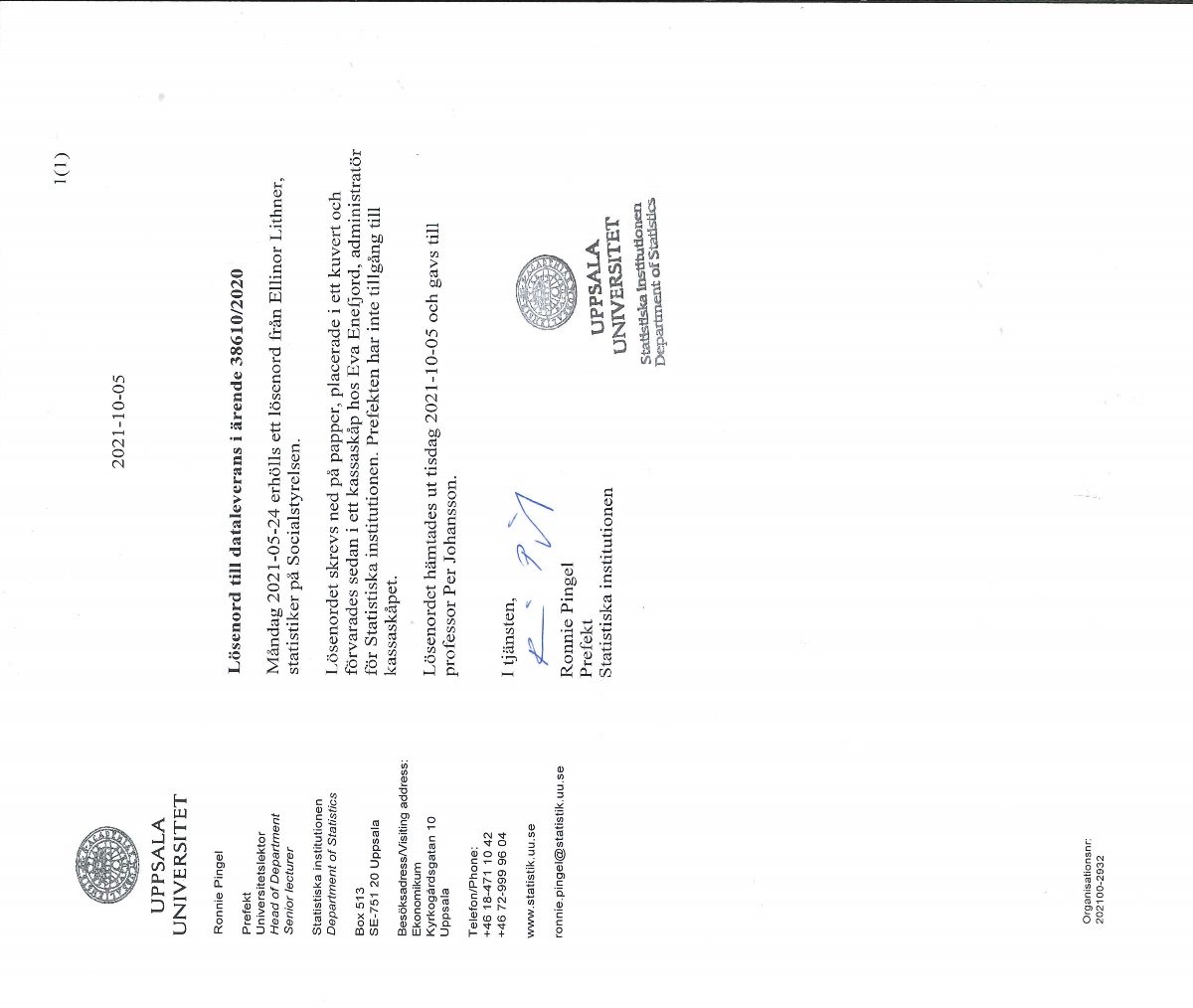
**S1 Text. Data delivery**

Translation: **Password for data delivery in lease 38610/2020**

Monday 2021-05-24 a password was received from Ellinor Lithner, statistician at the National Board of Health and Welfare.

The password was written down on paper, placed in an envelope and was then kept in a safe with Eva Enefjord, administrator for the Department of Statistics. The prefect does not have access to the cash cabinet.

The password was extracted on Tuesday 2021-10-05 and given to Professor Per Johansson.

In the service.

Ronnie Pingel,

Head of Department
