# Supplementary material for: Causal inferences and real-world evidence: A comparative effectiveness evaluation of abiraterone acetate against enzalutamide
Source: PLoS One. 2023 Oct 26;18(10):e0293000. doi: 10.1371/journal.pone.0293000 (PMC10602359; doi:10.1371/journal.pone.0293000)
Supplement: S2 Text — (DOCX) [file pone.0293000.s002.docx]

# S2 Text. Complementary analysis

## IV: First stage, validity test and sensitivity analysis.

**Table A: First stage probit regression**

| *Dependent variable: Prescription of AA* | |
| --- | --- |
| ALDER T | 0.024 (0.049) |
| I(ALDER Tˆ2) | −0.0001 (0.0003) |
| Civil0 | −2.100 (1.947) |
| Civil1 | −2.062 (1.948) |
| UTBNFORGYMN | −0.112^∗^ (0.060) |
| UTBNGYMN | −0.036 (0.055) |
| Diff time | 0.00002 (0.00002) |
| o tot1 | 0.040 (0.212) |
| f tot1 | −0.105 (0.105) |
| c tot1 | 0.481 (0.582) |
| d tot1:ALDER T | −0.003 (0.002) |
| d tot1:h tot1 | 0.098 (0.137) |
| h tot1:ALDER T | 0.001 (0.007) |
| c tot1:ALDER T | −0.005 (0.007) |
| SCORE D23 | −0.010 (0.050) |
| SCORE D24 | 0.075 (0.156) |
| MEDS C07 | 0.013 (0.052) |
| MEDS C08 | 0.041 (0.046) |
| MEDS A10 | −0.072 (0.101) |
| i211 | −0.239^∗∗∗^ (0.091) |
| i481 | 0.106 (0.065) |
| h tot ov1 | −0.150 (0.551) |
| Factor1 | 0.016 (0.020) |
| Factor2 | −0.003 (0.024) |
| Factor3 | −0.042 (0.029) |
| Factor4 | 0.032 (0.022) |
| Factor5 | −0.023 (0.028) |
| Factor6 | −0.075 (0.105) |
| Factor7 | 0.034 (0.024) |
| Factor8 | 0.015 (0.026) |
| Factor9 | 0.013 (0.027) |
| County:Dalarna | 0.528^∗∗∗^ (0.200) |
| County:Gavleborg | −0.077 (0.217) |
| County:Gotland | −0.154 (0.346) |
| County:Halland | 0.118 (0.209) |
| County:Jamtland | 0.516^∗∗^ (0.233) |
| County:Jonkopings lan | 0.117 (0.207) |
| County:Kalmar | 0.037 (0.223) |
| County:Kronoberg | 1.273^∗∗∗^ (0.204) |
| County:Norrbotten | 0.414^∗^ (0.213) |
| County:Orebro | 0.356^∗^ (0.207) |
| County:Ostergotlands lan | 0.071 (0.207) |
| County:Skane | −0.425^∗∗^ (0.204) |
| County:Sodermanland | −0.389 (0.250) |
| County:Stockholm | 0.176 (0.200) |
| County:Uppsala | 1.002^∗∗∗^ (0.197) |
| County:Varmland | 0.055 (0.216) |
| County:Vasterbotten | 0.516^∗∗^ (0.203) |
| County:Vasternorrland | 0.651^∗∗∗^ (0.208) |
| County:Vastmanland | 0.415^∗^ (0.214) |
| County:Vastra gotalands lan | 0.561^∗∗∗^ (0.181) |
| County specific mortality | −0.0005 (0.004) |

Standard errors within parentheses. ^∗^*p <*0.1; ^∗∗^*p <*0.05; ^∗∗∗^*p <*0.01.

**Table B: F-test from first stage probit regression, F-test of county factor.**

| First stage probit regression | Df | F statistic | p-value |
| --- | --- | --- | --- |
| County factor (*γ_C_*) | 20 | 16.24 | *<* 0*.*000 |

**Table C: Results from and F-tests of the county factor from a probit regression of SRE and Pain on covariates and the county factor.**

| Sensitivity analysis I, prevalence of SRE | Df | F statistic | p-value |
| --- | --- | --- | --- |
| County (*α*_1_) | 20 | 1.3526 | 0.1345 |
| Sensitivity analysis II, prevalence of pain | Df | F statistic | p-value |
| County (*α*_1_) | 20 | 1.0857 | 0.3567 |

Notes. Prevalence of SRE is an indicator that takes on value one if the individual has had one of the ICD codes specified between diagnosis and treatment. According to this definition, five percent of patients have had an SRE before treatment. Prevalence of pain is an indicator that takes on value one if the individual was prescribed opiates in combination with Paracetamol and Tramadol within three months, on one or more occasions between diagnosis and treatment. According to this definition, about three percent of patients have had severe pain before treatment.
