# Supplementary material for: Causal inferences and real-world evidence: A comparative effectiveness evaluation of abiraterone acetate against enzalutamide
Source: PLoS One. 2023 Oct 26;18(10):e0293000. doi: 10.1371/journal.pone.0293000 (PMC10602359; doi:10.1371/journal.pone.0293000)
Supplement: S3 Text — (DOCX) [file pone.0293000.s003.docx]

## S3 Text. Sub-analyses of SRE

The results from the sub-analyses are displayed in Figs S3-S5. The overall results are, to a large extent, the same as in the main analysis; there is a pattern of a negative effect with regard to SRE. The effect seems to be more apparent in the sub-samples of low-responders, patients with a long waiting time, and with a non-aggressive disease.


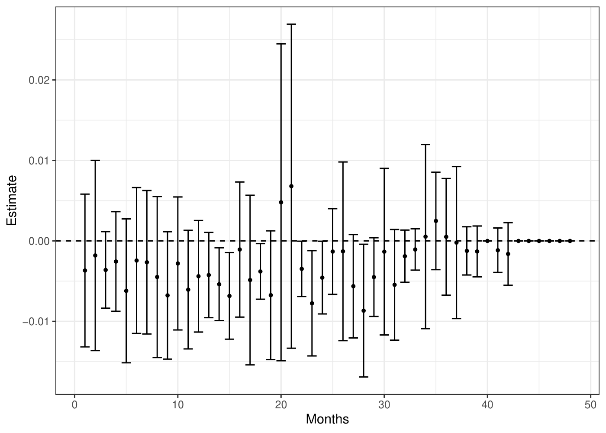

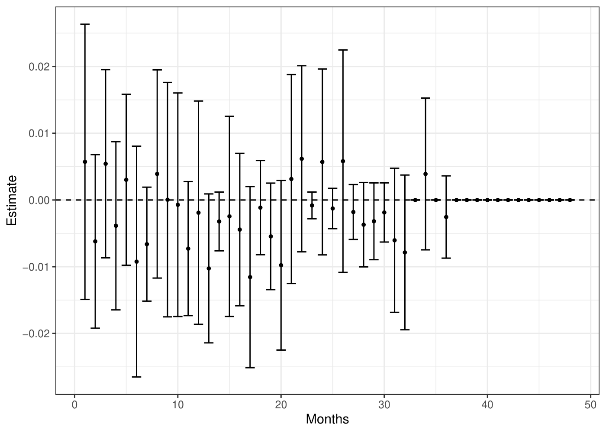


**Fig A: SRE. Estimates (ATE) and 95% Bonferroni corrected confidence intervals.** Left: low respondents, i.e. patients previously given hormone treatment for less than 12 months; right: high respondents, i.e. patients previously given hormone treatment for twelve months or more.


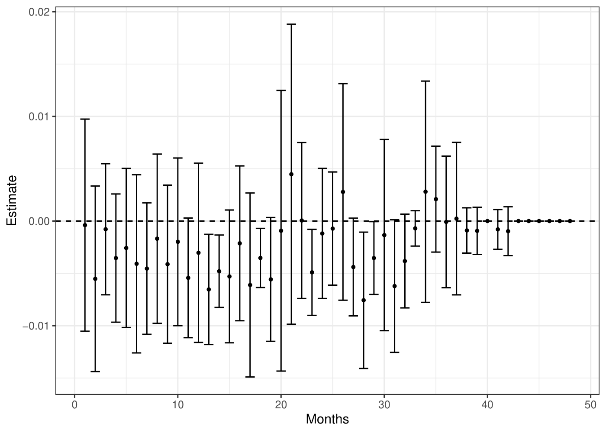

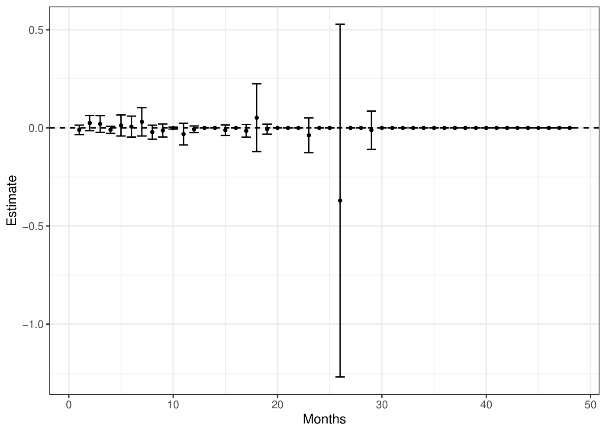


**Fig B: SRE. Estimates (ATE) and 95% Bonferroni corrected confidence intervals.** Left: non-aggressive disease, i.e. patients with no hospital record of visceral metastases before starting AA or ENZ treatment; right: aggressive disease, i.e. patients with hospital record of visceral metastases before starting AA or ENZ treatment.


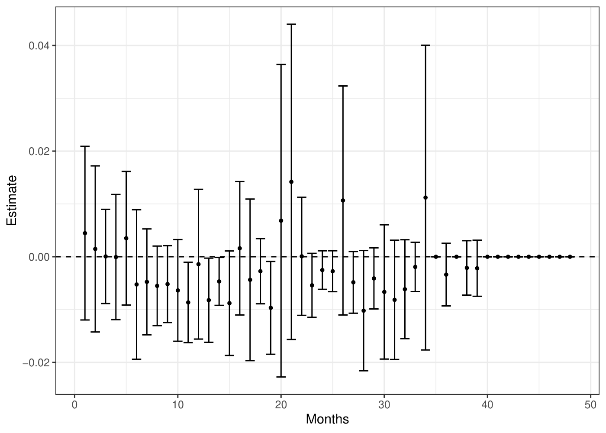

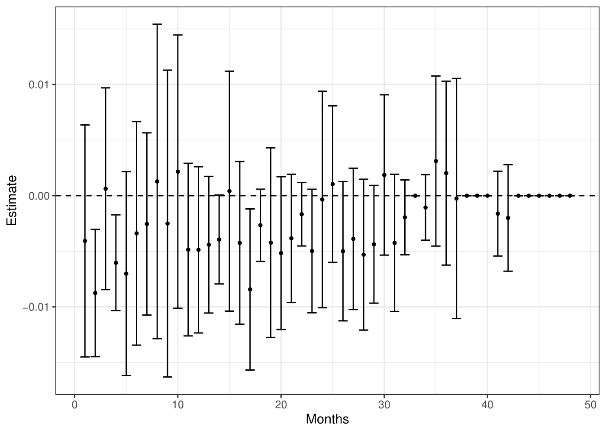


**Fig C: SRE. Estimates (ATE) and 95% Bonferroni corrected confidence intervals.** Left: long waiting time, i.e. patients having longer period of time between diagnosis of prostate cancer and prescription for AA or ENZ treatment compared to the median waiting time for both drugs. Right: short waiting time, i.e. patients having a shorter period of time between diagnosis of prostate cancer and prescription for AA or ENZ treatment compared to the median waiting time for both drugs.
