# Supplementary material for: Causal inferences and real-world evidence: A comparative effectiveness evaluation of abiraterone acetate against enzalutamide
Source: PLoS One. 2023 Oct 26;18(10):e0293000. doi: 10.1371/journal.pone.0293000 (PMC10602359; doi:10.1371/journal.pone.0293000)
Supplement: S4 Text — (DOCX) [file pone.0293000.s004.docx]

## S4 Text. Missing data

We run a regression on the indicator variable, taking on value one if missing and zero if not on the covariates used in the main analysis and the treatment indicator *W* = 1.

The results from the analysis are displayed in Table B4. From the table, we can see that there are 170, 437 and 1,176 missing observations for *SPSA*, *GleasSa* and *Mstad*, respectively. However, given the included covariates (see Fig 1), the level of missing data is not associated with the drug prescribed. Thus, in the following sensitivity analyses, patients with missing data are removed.

**Table A: Analysis of data from the NPCR and test for missing at random.**

|  | N missing | Estimate | SE | p-value |
| --- | --- | --- | --- | --- |
| *SPSA* | 170 | -0.01 | 0.006 | 0.3105 |
| *GleasSa* | 437 | 0.01 | 0.010 | 0.2530 |
| *Mstad* | 1176 | 0.01 | 0.015 | 0.5254 |

*OLS, inference with cluster robust standard errors*
