# Supplementary material for: Causal inferences and real-world evidence: A comparative effectiveness evaluation of abiraterone acetate against enzalutamide
Source: PLoS One. 2023 Oct 26;18(10):e0293000. doi: 10.1371/journal.pone.0293000 (PMC10602359; doi:10.1371/journal.pone.0293000)
Supplement: S5 Text — (DOCX) [file pone.0293000.s005.docx]

**S5 Text. Exploratory analysis**

Two exploratory endpoints are studied. The first is prostate-specific mortality and the second is compliance with treatment (COMPLY). COMPLY is a measurement of the duration until the patient is observed to have stopped the treatment. This duration is measured as the total number of daily doses collected, adjusted for compliance.

Prostate-specific mortality is measured in 30-day intervals in the same way as in the main analysis. The results are displayed in Fig A. The Fig displays the point estimates and the same Bonferroni corrected confidence intervals as in the main analysis.

All of the point estimates show an increased mortality from AA. Three of these point estimates are statistically significant (months 4, 7 and 8). All in all, this suggests that prostate-specific mortality may be higher if prescribed AA instead of ENZ.

A potential problem with these analyses is if death from other causes differs across the two drugs. The implication of this would be that the risk group differs between the two drugs. If, for instance, the ENZ patients have a higher mortality from other causes, then presumably ENZ patients at risk of dying have better health than AA patients at risk.

Assume that ENZ patients have a higher mortality from other causes and that those at risk have better health, then by treating all death from other causes as death from the prostate-cancer we get a “lower bound” of the comparative effect of AA against ENZ. If we treat those dying as alive (i.e. remaining at risk) we get an “upper bound” of prostate-specific mortality. The definition of “lower bound” and ‟upper bound” could be reversed if we instead assume the reverse pattern of death from other causes, and so should not be taken literally. Besides, if there is no significant difference between the upper and lower bounds, the results are interpreted as excess mortality of one drug in contrast to the other, and not a major problem with the main analysis on prostate-specific mortality.

The results from these analyses are displayed in Fig B. There are no clear differences in the results from the main analysis, stating a potentially higher prostate-specific mortality from prescribing AA instead of ENZ.





**Fig A: Prostate-specific mortality.** Estimates (ATE) and 95% Bonferroni corrected confidence intervals


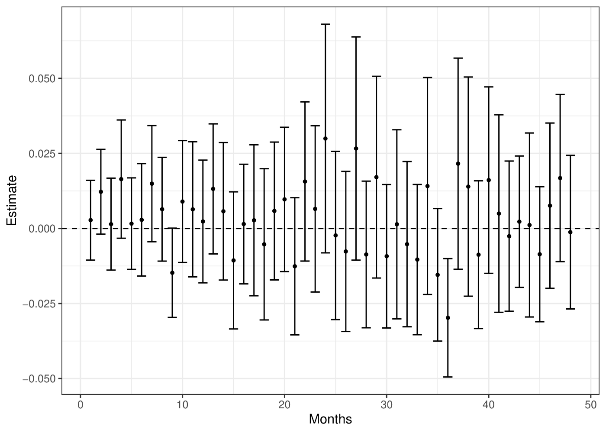

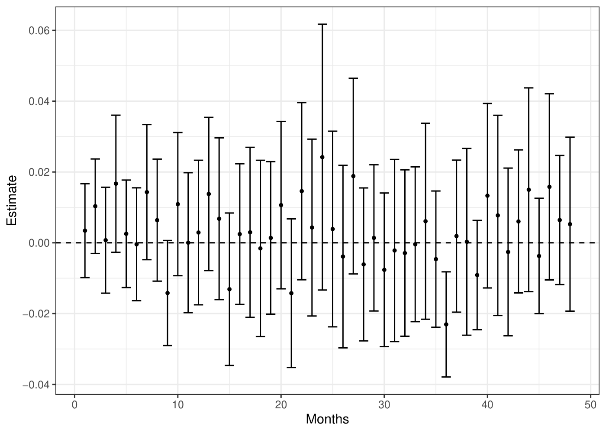


**Fig B: Bounds (given the presumption of more death from other causes in ENZ) of prostate-specific death.** Estimates and 95% Bonferroni corrected confidence intervals. Lower (left) and upper (right) bounds.

The analysis of COMPLY is conducted using Cox regression with covariates, competing event and clustering on individuals (R-package crrSC). The competing event is death. The results from the analysis are presented in Table A. Time on AA seems to be longer than on ENZ, but we find no statistically significant results.

Table A: Result for COMPLY, a measurement of the duration until the patient is observed to have stopped the treatment.

Estimate SE p-value

AA 0.03 0.05 0.52

*Note: Cox regression with competing events and cluster.*

The covariates included in the analysis are displayed in Table 1.
