# Supplementary material for: Causal inferences and real-world evidence: A comparative effectiveness evaluation of abiraterone acetate against enzalutamide
Source: PLoS One. 2023 Oct 26;18(10):e0293000. doi: 10.1371/journal.pone.0293000 (PMC10602359; doi:10.1371/journal.pone.0293000)
Supplement: S1 Table — (DOCX) [file pone.0293000.s006.docx]

**S1 Table. Included ICD codes and ATC codes.**

|  | **Cardiovascular disease** | **Diabetes** | **Osteoporosis** | **Metastases** | **Malaise and fatigue** |
| --- | --- | --- | --- | --- | --- |
| ICD codes | I21 | E10 | M859 | C77 | R53 |
|  | I22 | E11 | M810 | C78 |  |
|  | I252 | E12 | M818 | C79 |  |
|  | Z958 | E13 | M819 |  |  |
|  | Z959 | E14 |  |  |  |
|  | I70 |  |  |  |  |
|  | I71 |  |  |  |  |
|  | I731 |  |  |  |  |
|  | I738 |  |  |  |  |
|  | I739 |  |  |  |  |
|  | I771 |  |  |  |  |
|  | I790 |  |  |  |  |
|  | I792 |  |  |  |  |
|  | K551 |  |  |  |  |
|  | K558 |  |  |  |  |
|  | K559 |  |  |  |  |
|  | I090 |  |  |  |  |
|  | I110 |  |  |  |  |
|  | I13 |  |  |  |  |
|  | *I130* |  |  |  |  |
|  | *I131* |  |  |  |  |
|  | *I132* |  |  |  |  |
|  | *I139* |  |  |  |  |
|  | I42 |  |  |  |  |
|  | *I420* |  |  |  |  |
|  | *I421* |  |  |  |  |
|  | *I422* |  |  |  |  |
|  | *I423* |  |  |  |  |
|  | *I424* |  |  |  |  |
|  | *I425* |  |  |  |  |
|  | *I426* |  |  |  |  |
|  | *I427* |  |  |  |  |
|  | *I428* |  |  |  |  |
|  | *I429* |  |  |  |  |
|  | I43 |  |  |  |  |
|  | I44 |  |  |  |  |
|  | I45 |  |  |  |  |
|  | I46 |  |  |  |  |
|  | I47 |  |  |  |  |
|  | I48 |  |  |  |  |
|  | I49 |  |  |  |  |
|  | I50 |  |  |  |  |
|  | I51 |  |  |  |  |
|  | R00 |  |  |  |  |
|  |  |  |  |  |  |
| ATC codes | C07  C08 | A10 |  |  |  |
