# Supplementary material for: Causal inferences and real-world evidence: A comparative effectiveness evaluation of abiraterone acetate against enzalutamide
Source: PLoS One. 2023 Oct 26;18(10):e0293000. doi: 10.1371/journal.pone.0293000 (PMC10602359; doi:10.1371/journal.pone.0293000)
Supplement: S2 Table — (DOCX) [file pone.0293000.s007.docx]

S2 Table. Variable descriptions (* variable is included in factor analysis).

|  | Variable | | Description |  |
| --- | --- | --- | --- | --- |
|  | LOPNR | | ID serial number |  |
|  | group | | Treatment group (group=1 for abiraterone) |  |
|  | County | | County council |  |
|  | Diff time | | Time between diagnosis and treatment |  |
|  | SRE | | Skeleton-related event between diagnosis and treatment (SRE=1 if yes) |  |
|  | Pain | | Pain between diagnosis and treatment (Pain=1 if yes) |  |
|  | County specific mortality | | County specific mortality, the year of diagnosis |  |
|  | SCORE D22 | | Elixhouser index at diagnosis = 0 |  |
|  | SCORE D23 | | Elixhouser index at diagnosis = 1-4 |  |
|  | SCORE D24 | | Elixhouser index at diagnosis >= 5 |  |
|  | d_tot | | Indicator of diabetes before treatment |  |
|  | o_tot | | Indicator of osteoporosis before treatment |  |
|  | c_tot | | Indicator of metastases before treatment |  |
|  | i48 | | Indicator of atrial fibrillation and flutter before treatment |  |
|  | i21 | | Indicator of acute myocardial infarction before treatment |  |
|  | h tot ov i | | Indicator of other CVD before treatment |  |
|  | f tot | | Indicator of fatigue before treatment |  |
|  | h tot | | Indicator of any CVD before treatment |  |
| * | bfd 1m | | Number of visits, 1 month before diagnosis |  |
| * | bfd 12m | | Number of visits, 12 months before diagnosis |  |
| * | afd 1m | | Number of visits, 0-1 month after diagnosis |  |
| * | afd 2m | | Number of visits, 1-2 months after diagnosis |  |
| * | afd 3m | | Number of visits, 2-3 months after diagnosis |  |
| * | bft 1m | | Number of visits, 0-1 month before treatment |  |
| * | bft 2m | | Number of visits, 1-2 months before treatment |  |
| * | bft 3m | | Number of visits, 2-3 months before treatment |  |
| * | bft 4m | | Number of visits, 3-4 months before treatment |  |
| * | bft 5m | | Number of visits, 4-5 months before treatment |  |
| * | bft 12m tot | | Number of visits, 1 year before treatment |  |
| * | bft 12m tot c619 | | Number of C61.9 related visits, 1 year before treatment |  |
| * | msv days bd | | Number of days in inpatient care before diagnosis |  |
| * | msv days c619 | | Number of days in inpatient care related to C61.9 |  |
| * | msv tot prop c619 | | Fraction of days in inpatient care related to C61.9 |  |
| * | msv days | | Days in inpatient care between diagnosis and treatment |  |
| * | msv tot prop | | Fraction of days in inpatient care between diagnosis and treatment |  |
| * | bfd 60m | | Number of visits, 5 years before diagnosis |  |
| * | nn | | Visits per day between diagnosis and treatment |  |
| * | h tot bfd | | Visits related to CVD before diagnosis |  |
| * | h tot bw | | Visits related to CVD between diagnosis and treatment |  |
| * | d tot bfd | | Visits related to diabetes before diagnosis |  |
| * | d tot bw | | Visits related to diabetes between diagnosis and treatment |  |
| * | o tot bfd | | Visits related to osteoporosis before diagnosis |  |
| * | o tot bw | | Visits related to osteoporosis between diagnosis and treatment |  |
| * | c tot bfd | | Visits related to metastases before diagnosis |  |
| * | c tot bw | | Visits related to metastases between diagnosis and treatment |  |
| * | f tot bfd | | Visits related to fatigue before diagnosis |  |
| * | f tot bw | | Visits related to fatigue between diagnosis and treatment |  |
| * | i48 bfd | | Visits related to atrial fibrillation and flutter before diagnosis |  |
| * | i21 bfd | | Visits related to acute myocardial infarction before diagnosis |  |
| * | h tot ov bfd | | Visits related to other CVD before diagnosis |  |
| * | i48 bw | | Visits related to atrial fibrillation and flutter between diagnosis and treatment |  |
| * | i21 bw | | Visits related to acute myocardial infarction between diagnosis and treatment |  |
| * | h tot ov bw | | Visits related to other CVD between diagnosis and treatment |  |
| * | q1 t | Inpatient care visits between diagnosis and treatment, quartile 1 | | |
| * | q2 t | Inpatient care visits between diagnosis and treatment, quartile 2 | | |
| * | q3 t | Inpatient care visits between diagnosis and treatment, quartile 3 | | |
| * | q4 t | Inpatient care visits between diagnosis and treatment, quartile 4 | | |
|  | MEDS C08 | Number of prescriptions of ATC code C08 | | |
|  | MEDS C07 | Number of prescriptions of ATC code C07 | | |
|  | MEDS A10 | Number of prescriptions of ATC code A10 | | |
|  | ALDER T | Age at treatment | | |
|  | UTBNFORGYMN | Educational level at diagnosis: less than secondary school | | |
|  | UTBNGYMN | Educational level at diagnosis: secondary school | | |
|  | UTBNEFTERGYMN | Educational level at diagnosis: more than secondary school | | |
|  | Civil | Marital status at diagnosis (Civil=1 if partner) | | |
|  | Fodelseland EU28 | Country of birth | | |
| * | LoneInk | Wage income at treatment | | |
| * | InkFNetto | Income from business at treatment | | |
| * | KapInk | Capital income at treatment | | |
| * | DispInk | Disposable income at treatment | | |
| * | DispInkFam | Family disposable income at treatment | | |
| * | ForvErs | Earned income and work-related benefits at treatment | | |
| * | LoneInk 1y | Wage income one year before treatment | | |
| * | InkFNetto 1y | Income from business one year before treatment | | |
| * | KapInk 1y | Capital income one year before treatment | | |
| * | DispInk 1y | Disposable income one year before treatment | | |
| * | DispInkFam 1y | Family disposable income one year before treatment | | |
| * | ForvErs 1y | Earned income and work-related benefits one year before treatment | | |
| * | LoneInk 2y | Wage income two years before treatment | | |
| * | InkFNetto 2y | Income from business two years before treatment | | |
| * | KapInk 2y | Capital income two years before treatment | | |
| * | DispInk 2y | Disposable two years before treatment | | |
| * | DispInkFam 2y | Family disposable income two years before treatment | | |
| * | ForvErs 2y | Earned income and work-related benefits two years before treatment | | |
| * | SjukRe | Sickness compensation at treatment | | |
| * | ArbLos | Unemployment benefits at treatment | | |
| * | ForTid | Early retirement benefit at treatment | | |
| * | SocInk | Social security benefits at treatment | | |
| * | SocBidrPersF | Social security benefits at treatment | | |
| * | SocBidrFam | Social security benefits of the family at treatment | | |
| * | SjukRe 1y | Sickness compensation one year before treatment | | |
| * | ArbLos 1y | Unemployment benefits one year before treatment | | |
| * | ForTid 1y | Early retirement benefit one year before treatment | | |
| * | SocInk 1y | Social security benefits one year before treatment | | |
| * | SocBidrPersF 1y | Social security benefits one year before treatment | | |
| * | SocBidrFam 1y | Social security benefits of the family one year before treatment | | |
| * | SjukRe 2y | Sickness compensation two years before treatment | | |
| * | ArbLos 2y | Unemployment benefits two years before treatment | | |
| * | ForTid 2y | Early retirement benefit two years before treatment | | |
| * | SocInk 2y | Social security benefits two years before treatment | | |
| * | SocBidrPersF 2y | Social security benefits two years before treatment | | |
| * | SocBidrFam 2y | Social security benefits of the family two years before treatment | | |
| * | AldPens | Old-age pensions at treatment | | |
| * | SumTjP | Occupational pensions at treatment | | |
| * | PrivPens | Private pensions at treatment | | |
| * | AldPens 1y | Old-age pensions one year before treatment | | |
| * | SumTjP 1y | Occupational pensions one year before treatment | | |
| * | PrivPens 1y | Private pensions one year before treatment | | |
| * | AldPens 2y | Old-age pensions two years before treatment | | |
| * | SumTjP 2y | Occupational pensions two years before treatment | | |
| * | PrivPens 2y | Private pensions two years before treatment | | |
| * | LoneInk D | Wage income at diagnosis | | |
| * | InkFNetto D | Income from business at diagnosis | | |
| * | KapInk D | Capital income at diagnosis | | |
| * | DispInk D | Disposable income at diagnosis | | |
| * | DispInkFam D | Family disposable income at diagnosis | | |
| * | LoneInk 1y D | Wage income one year before diagnosis | | |
| * | InkFNetto 1y D | Income from business one year before diagnosis | | |
| * | KapInk 1y D | Capital income one year before diagnosis | | |
| * | DispInk 1y D | Disposable income one year before diagnosis | | |
| * | DispInkFam 1y D | Family disposable income one year before diagnosis | | |
| * | LoneInk 2y D | Wage income two years before diagnosis | | |
| * | InkFNetto 2y D | Income from business two years before diagnosis | | |
| * | KapInk 2y D | Capital income two years before diagnosis | | |
| * | DispInk 2y D | Disposable two years before diagnosis | | |
| * | DispInkFam 2y D | Family disposable income two years before diagnosis | | |
| * | SjukRe D | Sickness compensation at diagnosis | | |
| * | ArbLos D | Unemployment benefits at diagnosis | | |
| * | ForTid D | Early retirement benefit at diagnosis | | |
| * | SocInk D | Social security benefits at diagnosis | | |
| * | SocBidrPersF D | Social security benefits at diagnosis | | |
| * | SocBidrFam D | Social security benefits of the family at diagnosis | | |
| * | SjukRe 1y D | Sickness compensation one year before diagnosis | | |
| * | ArbLos 1y D | Unemployment benefits one year before diagnosis | | |
| * | ForTid 1y D | Early retirement benefit one year before diagnosis | | |
| * | SocInk 1y D | Social security benefits one year before diagnosis | | |
| * | SocBidrPersF 1y D | Social security benefits one year before diagnosis | | |
| * | SocBidrFam 1y D | Social security benefits of the family one year before diagnosis | | |
| * | SjukRe 2y D | Sickness compensation two years before diagnosis | | |
| * | ArbLos 2y D | Unemployment benefits two years before diagnosis | | |
| * | ForTid 2y D | Early retirement benefit two years before diagnosis | | |
| * | SocInk 2y D | Social security benefits two years before diagnosis | | |
| * | SocBidrPersF 2y D | Social security benefits two years before diagnosis | | |
| * | SocBidrFam 2y D | Social security benefits of the family two years before diagnosis | | |
| * | AldPens D | Old-age pensions at diagnosis | | |
| * | SumTjP D | Occupational pensions one year before diagnosis | | |
| * | PrivPens D | Private pensions two years before diagnosis | | |
| * | AldPens 1y D | Old-age pensions one year before diagnosis | | |
| * | SumTjP 1y D | Occupational pensions one year before diagnosis | | |
| * | PrivPens 1y D | Private pensions two years before diagnosis | | |
| * | AldPens 2y D | Old-age pensions two years before diagnosis | | |
| * | SumTjP 2y D | Occupational pensions two years before diagnosis | | |
| * | PrivPens 2y D | Private pensions two years before diagnosis | | |
|  |  |  | | |
