# Supplementary figures and images for: Causal inferences and real-world evidence: A comparative effectiveness evaluation of abiraterone acetate against enzalutamide
Source: PLoS One. 2023 Oct 26;18(10):e0293000. doi: 10.1371/journal.pone.0293000 (PMC10602359; doi:10.1371/journal.pone.0293000)

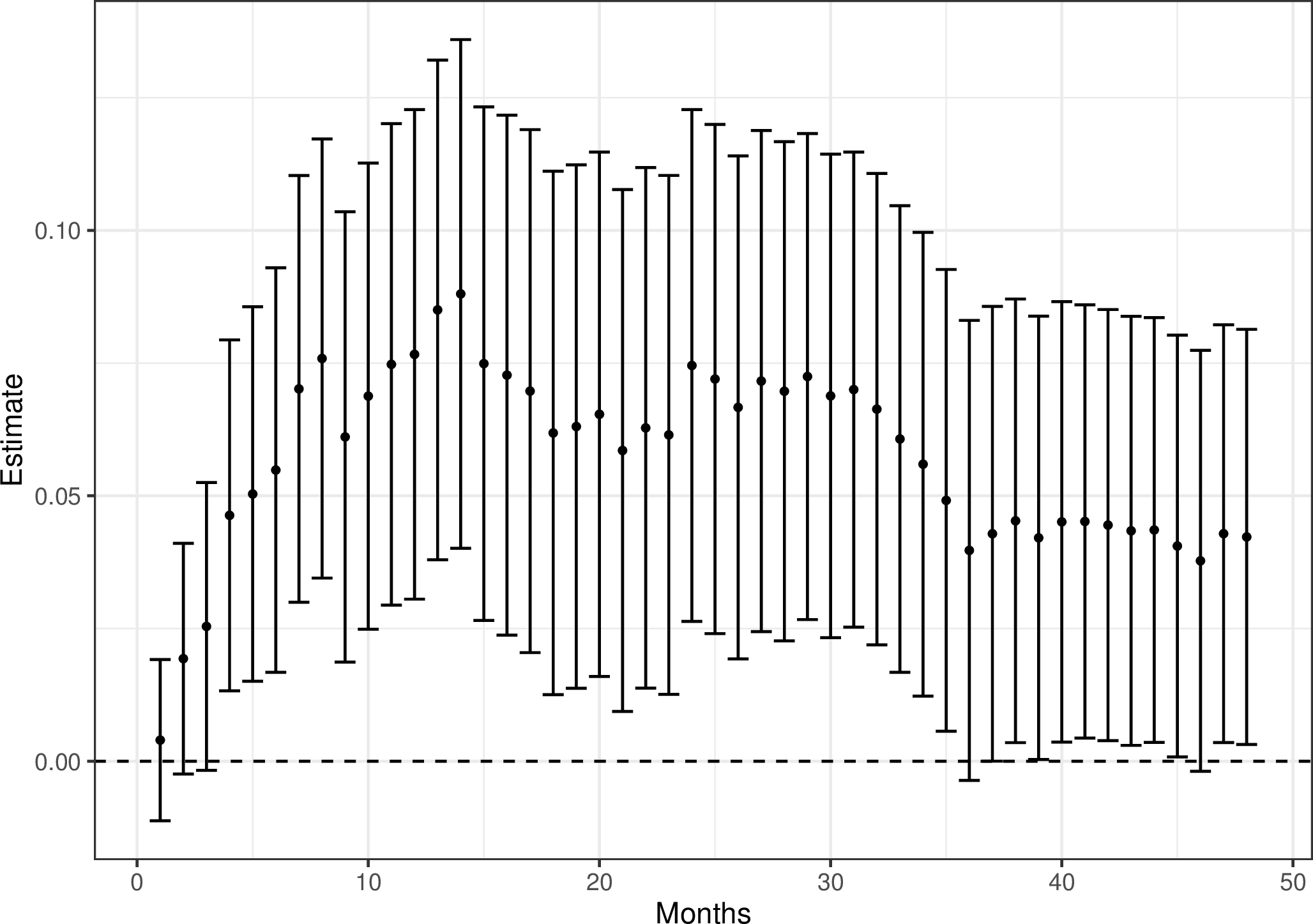

Supplement: S1 Fig — Excluding switchers (485 patients). (TIF) [file pone.0293000.s008.tif]
