# Supplementary material for: Causal inferences and real-world evidence: A comparative effectiveness evaluation of abiraterone acetate against enzalutamide
Source: PLoS One. 2023 Oct 26;18(10):e0293000. doi: 10.1371/journal.pone.0293000 (PMC10602359; doi:10.1371/journal.pone.0293000)
Supplement: Attachment — Submitted filename: Addressed Comments.doc [file pone.0293000.s012.doc]

|  | **Editor Comments:** | **Our comments:** |
| --- | --- | --- |
| 1 | Please ensure that your manuscript meets PLOS ONE's style requirements, including those for file naming. | We apologize for our failure to meets PLOS ONE’s style requirements, and have now made the below changes:   (1) We use Vancouver citing and referencing requirements (2) We have changed the format of the figures. (3) We have changed the caption of the figures and tables. (4) We have changed the font size of the headings. (5) We have moved the acknowledgments to the end.   However, as these changes are many, they have not been highlighted in yellow in the attached manuscript. |
| 2 | In your Data Availability statement, you have not specified where the minimal data set underlying the results described in your manuscript can be found. PLOS defines a study's minimal data set as the underlying data used to reach the conclusions drawn in the manuscript and any additional data required to replicate the reported study findings in their entirety. All PLOS journals require that the minimal data set be made fully available. Upon re-submitting your revised manuscript, please upload your study’s minimal underlying data set as either Supporting Information files or to a stable, public repository and include the relevant URLs, DOIs, or accession numbers within your revised cover letter. Any potentially identifying patient information must be fully anonymized.  Important: If there are ethical or legal restrictions to sharing your data publicly, please explain these restrictions in detail. Please see our guidelines for more information on what we consider unacceptable restrictions to publicly sharing data. Note that it is not acceptable for the authors to be the sole named individuals responsible for ensuring data access.  We will update your Data Availability statement to reflect the information you provide in your cover letter. | The micro data used for the analysis was created by combining several administrative registers held by Statistics Sweden, the National Board of Health and Welfare and the Swedish prostate cancer register. Access to this data is restricted by national and international regulations, and we cannot make the individual data public available (even anonymized). Therefore, we ask for an exemption to the data availability policy of this journal.  To assist researchers replicating the study we will make the ordering of the data available together with the code for analyses once our manuscript has been accepted for publication. The data need, however, be requested by the researchers themselves. The practical arrangements for accessing the data will, to some extent, depend on the location of the researcher. |
| 3 | Please include your full ethics statement in the ‘Methods’ section of your manuscript file. In your statement, please include the full name of the IRB or ethics committee who approved or waived your study, as well as whether or not you obtained informed written or verbal consent. If consent was waived for your study, please include this information in your statement as well. | As we describe the data in a separate section ‘Data’, we added the full name of the ethics committee. In addition, we described why written or verbal consent is waived for our study, see below and see lines 176-180:  The specific data we use and the way we process the data have obtained ethical approval from the Ethical Committee in Uppsala (ref. Dnr2017/482). Data are collected only from population registers administrated by the NBHW and SCB and quality registry administered by NPCR, which means that no informed consent was needed according to Swedish Act (2003:460) on Ethical Review of Research Involving Humans. |

|  | **Reviewer Comments:** | **Our comments:** |
| --- | --- | --- |
| 1 | Is the manuscript technically sound, and do the data support the conclusions?  The manuscript must describe a technically sound piece of scientific research with data that supports the conclusions. Experiments must have been conducted rigorously, with appropriate controls, replication, and sample sizes. The conclusions must be drawn appropriately based on the data presented.  Reviewer #1: No | As we received no negative feedback on methodology, we are unable to respond on the ‘No answer’.  We suspect the reviewer mistakenly responded ‘no’ instead of ‘yes’. The reason for this suspicion is that he/she responded yes on the question on “the statistical analysis been performed appropriately and rigorously?” Secondly, the reviewer writes “The authors present a novel approach to studying comparative effectiveness”. Furthermore, “These findings appear to be in agreement with prior indirect analyses of clinical trials which have shown comparatively improved treatment responses among patients receiving enzalutamide”. |
| 2 | Has the statistical analysis been performed äappropriately and rigorously?  Reviewer #1: Yes | No comment needed. |
| 3 | Have the authors made all data underlying the findings in their manuscript fully available?  The [PLOS Data policy](http://www.plosone.org/static/policies.action#sharing) requires authors to make all data underlying the findings described in their manuscript fully available without restriction, with rare exception (please refer to the Data Availability Statement in the manuscript PDF file). The data should be provided as part of the manuscript or its supporting information, or deposited to a public repository. For example, in addition to summary statistics, the data points behind means, medians and variance measures should be available. If there are restrictions on publicly sharing data—e.g. participant privacy or use of data from a third party—those must be specified.  Reviewer #1: Yes | No comment needed. |
|  | 4. Is the manuscript presented in an intelligible fashion and written in standard English?  PLOS ONE does not copyedit accepted manuscripts, so the language in submitted articles must be clear, correct, and unambiguous. Any typographical or grammatical errors should be corrected at revision, so please note any specific errors here.  Reviewer #1: Yes | No comment needed. |
|  | 5. A few important clarifications remain:  (1) Table 1 reveals that enzalutamide was the dominant therapy overall. Is there any data regarding regional factors influenced preferential prescription beyond subsidization? (2) Did the authors account for prior lines of therapy? Was it possible that patients receiving AA had previously received ENZ, and were therefore receiving this medication in the setting of treatment failure? (3) Other key variables to account for include PSA level at the time of treatment initiation, findings from bone scan (extent of disease). Were these accounted for?   Minor: -Figure captions are provided but the figure itself is not provided | (1) We have tried to understand the reason for the large regional variation by interviewing officials at TLV and doctors. The officials stated that it might be due to the negotiated agreements between the county councils, the drug company and the Pharmaceutical Benefits Board. Confronted with this proposition, the doctors agreed; however, they stated that it also could be due to differences in hospital recommendations or habits. Unfortunately, the agreements are confidential, which means that we cannot provide evidence on price variation. However, we did se variation in recommendation across the county councils. We have clarified this in the present version of the paper (Lines 218-225).  (2) At the time the drugs were introduced to the Swedish market, it was decided that patients would only receive one of these drugs under the reimbursement scheme. However, in our data, about 10% of the patients were prescribed both AA and ENZ over the years. We allocate these patients to the two samples, AA and ENZ-takers. This is the reason for why we say this is an intention to treat analysis.  We apologize for not having stating that “about 10% of the patients were prescribed both AA and ENZ over the years” and include this information in the present version of the paper (Line 204). However, this information and other details in the design of our paper, is summarized in extent in our two protocols published by BMJ Open and arXiv, see references in the manuscript.  (3) One aim of the paper/project was to evaluate the possibility of using only population registers in comparative effectiveness analysis. To this end, we did not use data from the prostate cancer register (NPCR) when we designed the study. However, even if we had used these data , we would not have been able to control for PSA at the time of prescription. NPCR only has information on the PSA level, Gleason Score, metastases, etc., at the time of diagnosis.  However, we presented the design to representatives of the drug companies and TLV before ordering the outcome data. We also asked about potential confounders not adjusted for in our design that are observed in NPCR. They agreed that the PSA level, Gleason Score, metastases at the time of diagnosis would be of interest to be used in an assessment of the validity of the study. Consequently, at the same time as ordering mortality data (after publishing the pre-analysis plan), we ordered data from the NPCR to assess the validity of the design. We found no difference between the two groups on these three variables. However, these tests are for uncounfoundness in general, so indirectly, also for PSA at the time of prescription.  In the present version of the paper, we have clarified that these variables are not possible to adjust for at the time of prescription. We write (lines 592-597)  “Thus, these sensitivity analyses provide quite strong evidence for the causal effects we find. One drawback of the sensitivity analyses is that these variables are measured at the time of diagnosis rather than at the time of prescription. Unfortunately, measurement at the time of prescription is not available in the NPCR data”. However, these tests are for uncounfoundness in general, so indirectly, also for PSA at the time of prescription.“  Minor:  We apologize for not providing the figures. The most likely reason was that we uploaded the wrong format of the figures. |
